# Commentary: Understanding the needs of individual families during crises such as the COVID‐19 pandemic—A commentary on Vogel et al. (2021)

**DOI:** 10.1111/jcv2.12012

**Published:** 2021-04-28

**Authors:** Lisa B. Thorell

**Affiliations:** ^1^ Department of Clinical Psychology Karolinska Institutet Stockholm Sweden

Vogel et al. (2021) investigated physical and psychological well‐being, as well as media use and peer relations/social support, at three time points: during 2019 (pre‐COVID baseline), the last week of March 2020 and during April 2020 (i.e., two time points shortly after the COVID‐19 lockdown). In addition, COVID‐19‐related feelings were assessed at the two time points during lockdown. The main findings were that peer relations/social support, as well as both physical and psychological well‐being, decreased from baseline to the time points during lockdown. Effects were significantly larger for children from families with low/medium socioeconomic status (SES). Only 20% of the children worried about getting infected by the virus themselves, while a majority worried about the situation with the COVID‐19 pandemic in general. The authors concluded that lockdown measures have to be balanced against adverse public health effects and that some children have been especially vulnerable to the negative effects of the COVID‐19 pandemic.

Children and adolescents seem to be less affected by COVID‐19 symptoms. However, studies such as the one by Vogel et al. (2021) have indicated that the effects of psychosocial impacts of the pandemic on children and adolescents have be relatively severe. This includes psychological and physical well‐being, which were studied by Vogel et al. (2021), as well as more specific behavioral problems such as irritability/aggression, inattention, and internalizing problems investigated in other studies (e.g., Orgiedes et al., 2020). In addition, many adults' work situation has been negatively affected (e.g., temporary layoffs or even job loss), especially among those with low‐/middle‐income jobs. It has also been emphasized that economic crises such as those caused during the COVID‐19 pandemic are related to a range of negative effects on parents, such as mental health problems, family conflict, and increased alcohol consumption, which in turn could lead to domestic violence and child maltreatment (e.g., Fegert et al., 2020).

Even though we know from previous reports that the COVID‐19 pandemic has had negative impacts on many different aspects of daily life functioning, few studies have included longitudinal data. Thus, Vogel et al. (2021) should be commended for taking advantage of data collected within the LIFE Child‐study prior to the pandemic and then for collecting additional data during two time points in the early phase of the lockdowns. Another advantage is the use of self‐ratings rather than parent ratings to assess well‐being and peer relations/support. However, because some of the data were collected before the pandemic, the scales used were not selected because they are the best measures for investigating the effects of a crisis, but rather for studying development in general. I believe that studies using general scales such as the KIDSCREEN, which Vogel et al. (2021) used, or the Strength and Difficulties Questionnaire, which has been used in other COVID‐19 studies, might underestimate the negative effects. The reason for this is that these questionnaires do not include questions regarding the specific aspects on daily life functioning that have necessarily been most affected during the COVID‐19 pandemic. Naturally, however, a large advantage of using standardized questionnaires is that the data collected during the pandemic can be compared with normative data collected under normal circumstances.

It should also be noted that, despite the fact that the KIDSCREEN might not fully capture difficulties specifically related to the COVID‐19 pandemic, the negative effects reported by, for example, Vogel et al. (2021) are relatively large. This was found despite the fact that families with low SES were underrepresented in their study as well as in several other questionnaire studies conducted during the pandemic. Thus, the psychosocial effects on children and adolescents have most likely been even more severe than what has been shown in the research published thus far.

One of the most important changes for many children and adolescents is the fact that most countries have closed down schools during at least part of the pandemic. We ( Thorell et al., in press) conducted a large‐scale study including seven European countries, which investigated the effects of distance education (often also referred to as homeschooling) during the COVID‐19 pandemic. Results showed that a relatively large proportion of parents reported negative effects of distance education on their children (e.g., finding it impossible to get distance education to work well, not being able to fully participate in distance education), on themselves (e.g., parental stress/worry), and on social relations within the family (e.g., increased conflicts between parents or between the parents and the child). Some differences between the seven countries were found. However, these differences were generally small, indicating that many parents across countries reported negative experiences.

Vogel et al. (2021) concluded that some children have been especially vulnerable to the negative effects of the COVID‐19 pandemic. If we are to face the next crisis better than we have dealt with the COVID‐19 pandemic, I believe we need more research focused on how we can best provide support for these families. In our study (Thorell et al., in press), parents of children with mental health problems generally reported more negative effects of distance education compared to parents of children without mental health problems. These findings are in line with several other studies emphasizing that children with neuropsychiatric disorders, such as Attention Deficit Hyperactivity Disorder (ADHD), might be particularly vulnerable to the negative effects of the pandemic (e.g., Becker et al., 2020) and recommendations for how to best continue the assessment and treatment of ADHD during the pandemic have therefore been presented (e.g., Cortese et al., 2020).

With regard to support for vulnerable groups during the pandemic, it is important to remember that disorders such as ADHD are highly heterogeneous and that support should therefore be provided based on individual needs. It is well‐known that executive deficits are strongly related to academic achievement. In our study on distance education during the COVID‐19 pandemic, we have now conducted additional analyses showing significantly larger negative effects on children diagnosed with ADHD who have executive deficits, than on both children with ADHD who do not have these deficits and children without any mental health problems (Thorell, 2021). These findings are not surprising given that distance education likely puts increased demands on executive functioning. For example, during online teaching, students are often required to use unfamiliar computer applications in addition to learning new academic content. Students are also required, to a much greater extent, to plan their own schoolwork during distance education compared to what is required during a normal school day. Because of these higher demands, research has shown that distance education has created a new subgroup of children (including those with and without ADHD) who did not have special educational needs before the pandemic, but whose parents report that they have been in need of such support during the pandemic ( Thorell et al., in press).

Although it may be argued that digital devices can be an important tool in educational settings, perhaps especially for students with special educational needs, previous research has also identified a number of potential problems. For example, a study on the use of digital devices in a normal school setting (Kay et al., 2017) showed that students were likely to become distracted, to have trouble initiating/completing tasks, getting engaged in off‐task media, or to try unsuccessfully to multitask. When the students themselves were asked how digital media use could best be improved, the most common answer was that stricter rules and supervision by teachers was necessary. This is of course difficult, or even impossible, for teachers to achieve when students are engaged in distance education. Several studies have also shown that many children and adolescents have greatly increased their use of digital media for non‐school‐related activities during the pandemic ( Thorell et al., in press; Vogel et al., 2021).

The fact that students themselves feel that rules and external supervision are needed for them to be able to focus on their studies points to the importance of parental support during distance education. In our study (Thorell et al., in press), we found that only about 14% of the time spent on school activities each day was spent in contact with a teacher (i.e., online webinars), with 48% being spent on self‐study and 30% in contact with a parent. However, relatively large differences between countries were found, and Germany, the country in which Vogel et al. (2021) conducted their study, was one of the countries with the lowest level of online teaching (only 5% on average and most children did not receive any online teaching at all). This has of course been a great burden for parents, especially those with limited resources. Vogel et al. (2021) found that the decrease in psychological well‐being, physical well‐being and peer relations/social support was more pronounced in families with medium/low SES. As mentioned above, families with low SES were underrepresented in the study by Vogel et al. (2021), as well as in most other studies investigating the psychosocial effects of the pandemic. Further studies including these families are essential, as previous research has shown that even during summer vacation, the well‐being of children from low‐SES families can be compromised in terms of access to healthy food, personal safety, and emotional support (e.g., Sepúlveda, 2020).

Finally, I would like to emphasize that, although a relatively large number of studies have reported negative effects on psychosocial outcomes for children and adolescents during the pandemic, we still have no knowledge about the long‐term consequences. The extensive time period under severe lockdown has most likely created greater differences in learning among students in the same class, making it very challenging for teachers to meet the needs of individual students when schools reopen after the pandemic.

In sum, I agree with the conclusion drawn by Vogel et al. (2021), which is that lockdown measures have to be balanced against adverse public health effects. It is clear from the research conducted thus far, that some children have been especially vulnerable to the negative effects of the COVID‐19 pandemic, but it is important that we do not base support solely on what type of diagnosis the child has. Instead, a more holistic view needs to be applied, which takes into consideration factors such as the child's underlying neurocognitive deficits (i.e., poor executive functioning) and the level of parental support. Finally, I hope that researchers like Vogel et al. (2021) plan on collecting additional data once the lockdowns are over. Such studies should be able to provide important information on the more long‐term effects of the pandemic on children and adolescents.

## CONFLICT OF INTEREST

The author has declared that they have no competing or potential conflicts of interest.
